# Inhibition of Integrin *α*v*β*6 Activation of TGF‐*β* Attenuates Tendinopathy

**DOI:** 10.1002/advs.202104469

**Published:** 2022-02-15

**Authors:** Xiao Wang, Shen Liu, Tao Yu, Senbo An, Ruoxian Deng, Xiaohua Tan, Janet Crane, Weixin Zhang, Dayu Pan, Mei Wan, Andrew Carr, Xu Cao

**Affiliations:** ^1^ Department of Orthopaedic Surgery The Johns Hopkins University School of Medicine Baltimore MD 21205 USA; ^2^ Nuffield Department of Orthopaedics Rheumatology and Musculoskeletal Sciences University of Oxford Oxford OX3 7LD UK

**Keywords:** integrin *α*v*β*6, tendinopathy, transforming growth factor‐beta

## Abstract

Tendinopathy is a common tendon disorder that causes pain and impairs function. It is the most common reason for consultation with musculoskeletal specialists. The available therapies for tendinopathy are limited in number and efficacy and have unclear cellular and molecular mechanisms. Here it is shown that transforming growth factor‐beta (TGF‐*β*) activated by integrin *α*v*β*6 promotes tendinopathy in mice. Excessive active TGF‐*β* is found during tendinopathy progression, which led to tenocytes’ phenotype transition to chondrocytes. Transgenic expression of active TGF‐*β* in tendons induced spontaneous tendinopathy, whereas systemic injection of a TGF‐*β* neutralizing antibody attenuated tendinopathy. Inducible knockout of the TGF‐*β* type 2 receptor gene (Tgfbr2) in tenocytes inhibited tendinopathy progression in mice. Moreover, it is found that integrin *α*v*β*6 induces TGF‐*β* activation in response to mechanical load in tendons. Conditional knockout of the integrin *α*v gene in tendons prevented tendinopathy in mice. The study suggests that integrin *α*v*β*6 activation of TGF‐*β* is the mechanism of tendinopathy, and that integrin *α*v*β*6 may be a therapeutic target in tendinopathy.

## Graphical abstract

In the context of physiological load, the latency‐associated protein (LAP) is linked with tenocytes via arginine‐glycine‐ascorbate amino acid (RGD)‐*α*v*β*6 associate, and TGF‐*β* remains latent. Excessive or repetitive loading induces conformation changes of LAP, leading to the release of active TGF‐*β* which binds to its receptor and initiates proliferation, apoptosis, and chondrogenesis of tenocytes as well as excessive vessel formation in tendons.

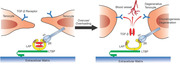

## 1 Introduction

During the past 3 decades, as more people participate in sports, the incidence of musculoskeletal diseases and the costs to treat them have increased, contributing to the rapidly increasing economic impact of musculoskeletal conditions.^[^ ^1^ ^]^ The cost of musculoskeletal disorders in the U.S. increased as a share of gross domestic product from 3.4% in 1996 to 5.8% in 2014, exceeding $900 billion in 2014.^[^ ^1a^ ^]^ Tendinopathy is a common tendon disorder that causes pain and impairs function, normally caused by overuse/overload.^[^ ^1^, ^2^ ^]^ It is the most common reason for consultation with a musculoskeletal specialist.^[^ ^1^, ^2^, ^3^ ^]^ However, the cellular and molecular mechanisms that drive the development of tendinopathy remain unclear, and no effective disease‐modifying treatment for tendinopathy exists.^[^ ^1b^ ^]^

Tendon is a tough band of fibrous connective tissue that connects muscle to bone. Collagens are the primary content of the extracellular matrix (ECM) of tendon tissue. The most abundant form of collagen is type‐1 collagen (Col1),^[^ ^4^ ^]^ while cartilage/chondrocyte specific type‐2 collagen (Col2) is rarely seen.^[^ ^5^ ^]^ Additionally, type‐3 collagen (Col3), which dominates early phases of wound healing and granulation tissue formation, is also very low in tendons. Tenocytes are spindle‐like, fibroblast‐type cells with elongated nuclei and are the major cell type residing in tendon. Tenocytes are responsible for synthesis and turnover of collagen and ground substance, such as proteoglycan. Scleraxis (Scx) is a transcription factor specific for tenocytes and tendon stem/progenitor cells (TSPCs) and is essential for tendon development.^[^ ^6^ ^]^ Scx‐deficient mice show a complete loss of major force‐transmitting and intermuscular tendons.^[^ ^7^ ^]^ Thus, despite the relatively lower expression in mature tenocytes than that in embryonic cells, Scx has been used to identify tenocytes at all stages of their development^[^ ^8^ ^]^ and ideal for analyzing tendon tissue homeostasis and pathology.^[^ ^9^ ^]^

Whether inflammation exists tendinopathic tendon is still in debate.^[^ ^1^, ^10^ ^]^ Disrupted collagen fibers are present in tendinopathic tendons.^[^ ^11^ ^]^ The number of tenocytes is increased, with prominent, rounded nuclei without a spindle‐like shape.^[^ ^12^ ^]^ Tenocytes located at the site of tendinopathy produce increased concentrations of proteoglycans,^[^ ^13^ ^]^ which may lead to chondrogenic differentiation.^[^ ^14^ ^]^ Changes in the expression and activity of various matrix‐degrading enzymes occur in tendinopathy, particularly matrix metalloproteinase 13 (MMP13).^[^ ^15^ ^]^ Apoptotic tenocytes are present in injured tendons and may result in massive cell death.^[^ ^12^, ^16^ ^]^ In degenerative tendinopathy, the aberrant formation of blood vessels, a process that can be associated with tendon repair or chronic pain,^[^ ^17^ ^]^ is present under ultrasonography^[^ ^18^ ^]^ and confirmed histologically.^[^ ^19^ ^]^

Transforming growth factor‐beta (TGF‐*β*) is expressed with the latency‐associated protein, rendering it inactive by masking the binding side of TGF‐*β*.^[^ ^20^ ^]^ Although latent TGF‐*β* is stored in large amounts in tendon and tenocytes have TGF‐*β* receptors, TGF‐*β* requires force to be activated.^[^ ^21^ ^]^ Heinemeier et al.^[^ ^22^ ^]^ reported that the mRNA expression of TGF‐*β*1 in tendon was markedly increased by different physical training in rats. It is also reported that mechanical force could induce active TGF‐*β* secretion in adult tendon homeostasis.^[^ ^6a^ ^]^ Precise activation of TGF‐*β* is essential to maintain cell function and homeostasis, while discontinuity of the temporal and spatial activation of TGF‐*β* leads to skeletal complications, including genetic bone diseases such as Camurati–Engelmann disease (CED), and more common musculoskeletal disorders such as enthesopathy, heterotopic ossification, osteoarthritis and degenerative disc disease.^[^ ^23^ ^]^ Integrins are dimeric cell surface receptors composed of *α* and *β* subunits.^[^ ^24^ ^]^ Recent studies found that integrins play a role in TGF‐*β* activation^[^ ^25^ ^]^ during myofibroblast contraction, which requires the binding of integrin *α*v*β*6 to an arginine‐glycine‐ascorbate amino acid (RGD) sequence in the prodomain of TGF‐*β* and exertion of force on this domain.^[^ ^26^ ^]^ Therefore, integrin *α*v*β*6 could induce a TGF‐*β* activating conformational change in response to force.^[^ ^27^ ^]^ On the basis of these studies, it is logical to postulate that activation of TGF‐*β* via integrin *α*v*β*6 in tendon by mechanical imperfection is involved in the pathogenesis of tendinopathy.

In this study, we found excessive active TGF‐*β*1 and accumulation of degenerative tenocytes in the tendons of tendinopathy patients consistent with previous studies. In our tendinopathy mouse models, we also found high levels of active TGF‐*β*1 during the progression of tendinopathy. Tenocytes gradually changed from having elongated nuclei to rounded ones, they produced proteoglycan over time, and they gained expression of Col2 suggesting phenotype transition to chondrocytes. Overexpression of active TGF‐*β*1 in tendons induced spontaneous tendinopathy. Systemic inhibition of TGF‐*β* activity by injection of a TGF‐*β* neutralizing antibody or knockout of the TGF‐*β* type 2 receptor gene (Tgfbr2) in tenocytes efficiently blocked progression of tendinopathy. Furthermore, a specific integrin, *α*v*β*6, activated latent TGF‐*β* during tendinopathy progression. Inhibition of TGF‐*β* activation by knocking out the Itgav gene in tenocytes attenuated tendinopathy progression.

## 2 Results

### 2.1 TGF‐*β*1 Activity is Elevated in Human Tendinopathy Tissue

**Figure 1:**
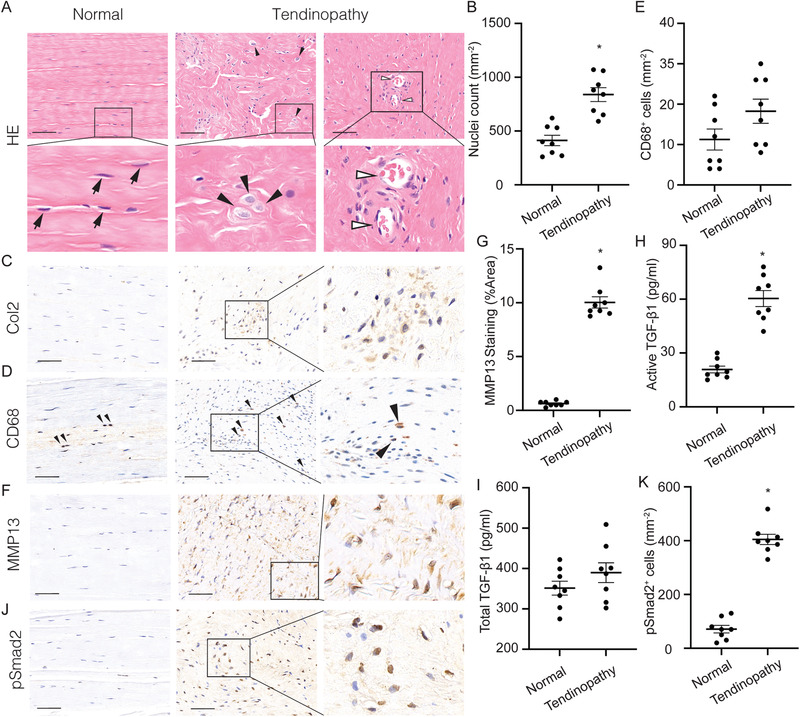
Elevated active transforming growth factor‐beta (TGF‐*β*) levels in human tendinopathy. A) HE staining of normal tendon (left) and tendinopathic tendon (middle and right) and B) quantification of nuclei counts per bone marrow area (mm^2^). Black arrows, tenocytes; black arrowheads, chondrocyte‐like cells; open arrowheads, blood vessels. Second row of A) shows high magnification of boxed area in first row. C) Immunostaining of type‐2 collagen (Col2) in both normal and tendinopathic tendons. D,F) Immunostaining and E,G) quantification and D,E) CD68^+^ cells and F,G) MMP13^+^ staining area per tissue area (mm^2^). Black arrowheads, CD68^+^ macrophages. H,I) Quantification of H) active and I) total TGF‐*β*1 in tendons. J) Immunostaining and K) quantification of pSmad2^+^ cells per tissue area (mm^2^). Scale bars: 200 µm. All data are shown as the mean ± standard deviation (*n* = 8 per group). *p < 0.05 compared with normal tendon as determined by unpaired, 2‐tailed Student's *t*‐test.

To determine the pathogenesis of tendinopathy, we examined surgical specimens of tendinopathic Achilles tendons. Healthy hamstring (semitendinosis) tendons were used as controls. Hematoxylin and eosin (HE) staining of the tendon specimens revealed that cell numbers were significantly increased in the tendinopathic tendons compared with normal controls (**Figure** **1**A,B). The spindle‐like nuclei in normal tenocytes had changed to rounded, chondrocyte‐like nuclei (Figure 1A). Immunostaining of Col2 confirmed chondrocytes formation in tendinopathic tendons (Figure 1C). The elongated fibers in the normal tendons were disrupted and disorganized in tendinopathy (Figure 1A middle panel). Moreover, an increase in vascularity at the site of tendinopathy was present compared with normal, hypovascular tendon (Figure 1A right panel). Slightly more inflammatory cells infiltrated in tendinopathic tendons, as shown by CD68 staining, but failed to reach statistical significance, suggesting that inflammation did not play a major role (Figure 1D,E). MMP13 staining revealed that significantly more MMP13 production was present in tendinopathy compared with healthy tendons, revealing a degenerative process (Figure 1F,G). Interestingly, we found significantly elevated levels of active TGF‐*β*1 in the tendinopathic tendons relative to the normal tendons (Figure 1H), while no significances were noted in total TGF‐*β*1 concentration (Figure 1I). We further tested the expression of the canonical TGF‐*β*1 downstream signaling transducer, phosphorylated Smad2 (pSmad2). The number of pSmad2^+^ cells was significantly greater in tendons with tendinopathy compared with normal tendons (Figure 1J,K). Altogether, our results reveal that tendinopathy in humans progresses via degeneration with high levels of active TGF‐*β*1 in the microenvironment.

### 2.2 Excessive TGF‐*β*1 is Activated in the Tendon of a Mouse Tendinopathy Model

**Figure 2:**
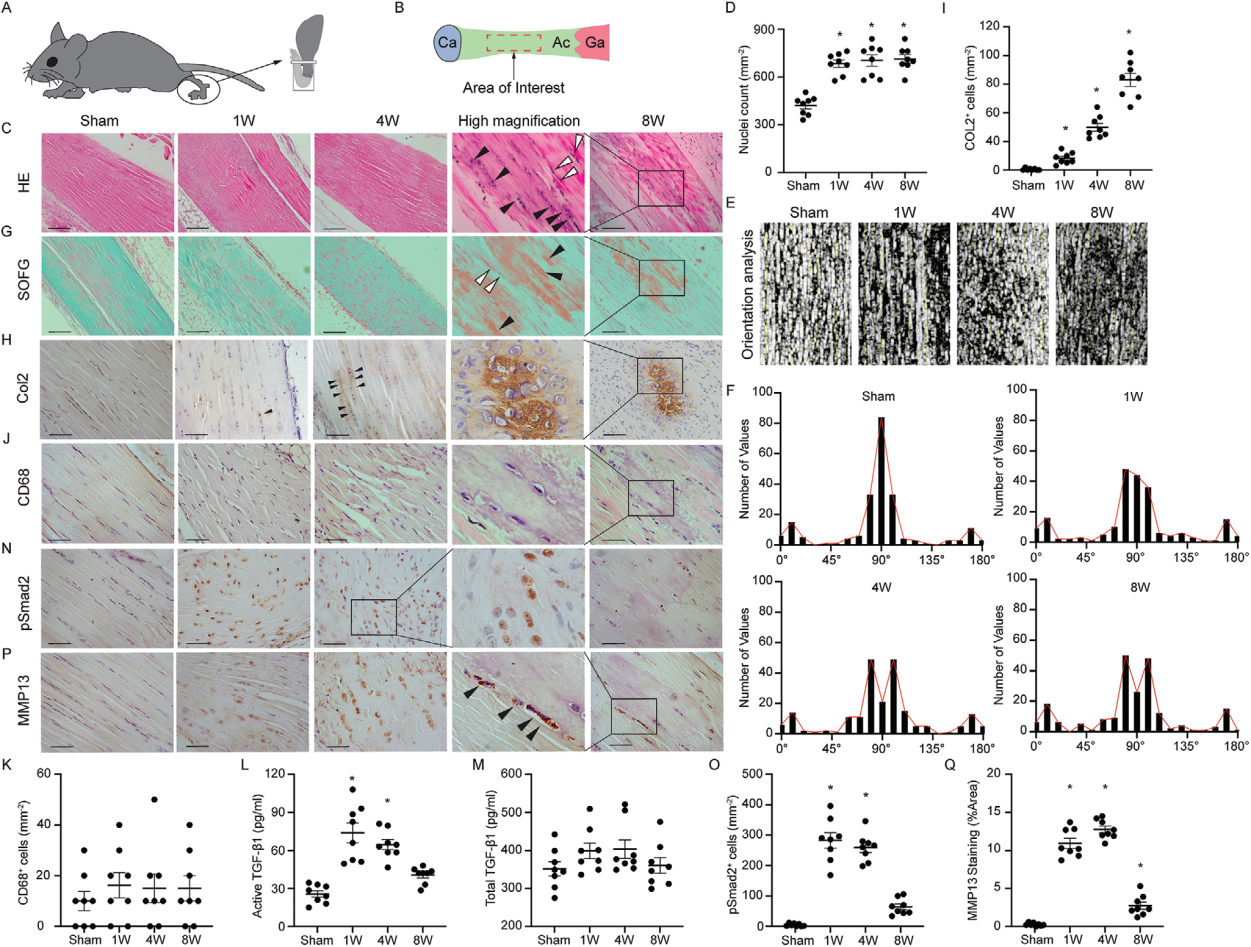
Elevated active TGF‐*β* levels are associated with tendinopathy in mice. A) A schematic diagram of the DI model. DI devices are applied to the right feet of mice to maintain dorsiflexion for 12 h each day for 1, 4, and 8 weeks as noted in results. Non‐immobilized littermates without devices are used as controls and allowed to move freely in the cages. B) Illustrations of observed area. The mid‐portion of Achilles tendon (Ac) is used as an area of interest (boxed area) from 2 to 6 mm proximal to the calcaneus (Ca). Ga, Gastrocnemius. (C, G, H, J, N, and P) Staining of sham‐processed tendon (first column) or tendons 1 week (second column), 4 weeks (third column), and 8 weeks (fifth column) after tendinopathy induction by DI. The fourth column is the boxed area of graphs of either 4 or 8 weeks after DI in high magnification. C) HE staining of normal tendon and tendinopathic tendons. Black arrowheads, chondrocyte‐like cells with rounded nuclei; open arrowheads, normal tenocytes with spindle‐like nuclei. Scale bar: 200 µm. D) Quantification of nuclei after sham or DI processing. E) The orientation of collagen fibers is analyzed using an OrientationJ plugin of ImageJ by evaluating every patch of the image (Yellow segments). F) Frequency distribution of orientation degree. (G) SOFG staining of normal tendon and tendinopathic tendons. Proteoglycan (red) and collagenous matrix (green). Black arrowheads, chondrocyte‐like cells; open arrowheads, normal tenocytes. Scale bar: 200 µm. H) Immunohistochemical staining and I) quantitative analysis of Col2^+^ chondrocytes per tissue area (mm^2^) in Achilles tendons. Black arrowheads, chondrocytes. Scale bar: 100 µm. J,N,P) Immunohistochemical staining and K,O,Q) quantitative analysis of J,K) CD68^+^ macrophages, N,O) pSmad2^+^ cells, and P,Q) MMP13^+^ area per tissue area (mm^2^) in Achilles tendons. Black arrowheads, MMP13^+^ cells. Scale bar: 50 µm. L,M) The concentration of L) active and M) total TGF‐*β*1 in Achilles tendon in sham and DI mice. All data are shown as the mean ± standard deviation (*n* = 8 mice per group). *p < 0.05 compared with sham operated mice as determined by one‐way analysis of variance to determine significance between groups.

To examine the progression of tendinopathy, we generated two tendinopathy models based on theories that tendinopathy results from tensile overload or overuse.^[^ ^28^ ^]^ Because Achilles tendinopathy is one of the most frequent form of tendinopathy,^[^ ^29^ ^]^ we focused on this particular tendon. For tensile overload model, we created an Achilles tendinopathy model, termed “dorsiflexion immobilization” (DI), in which we fixed the ankles of mice in dorsiflexion to mimic the overstrain of Achilles tendons with increased mechanical loading (**Figure** **2**A) and observed the mid‐portion of Achilles tendons (Figure 2B).^[^ ^23b^ ^]^ HE staining showed that cell numbers in the Achilles tendon were significantly increased at 1, 4, and 8 weeks after DI compared with sham‐operated controls (hereafter, “control mice”) (Figure 2C, D). Instead of the clearly defined and parallel collagen bundles seen in control mice, tendons in DI mice showed loss of the longitudinal alignment of collagen fibers and the clear demarcation between adjacent collagen bundles gradually (Figure 2E,F). Safranin‐O and fast green (SOFG) staining revealed evident proteoglycan deposition 8 weeks after tendinopathy induction, suggesting a disorganized ECM (Figure 2G). Immunostaining of Col2 showed that the deposition of Col2 increased gradually from 1 week after DI, with evident cartilage formation at week 8 (Figure 2H,I). CD68 staining showed few CD68^+^ cells in the Achilles tendon after DI, suggesting that inflammation did not play a major role in DI induced tendinopathy (Figure 2J,K). The concentration of active TGF‐*β*1 was higher in tendons after DI compared with tendons in control mice (Figure 2L), while the level of total TGF‐*β*1 did not change (Figure 2M). The increased active TGF‐*β*1 in the tendinopathic tendons was validated through immunohistochemistry analysis with elevated numbers of pSmad2^+^ cells after DI compared to sham controls (Figure 2N,O). We also observed an elevated production of MMP13 in the DI tendons compared with those in control mice, suggesting a degenerative process (Figure 2P,Q). Furthermore, blood vessels invaded the Achilles tendon after DI and increased gradually during the development of tendinopathy (Figure S1A,B, Supporting Information).

To further investigate the pathogenesis of tendinopathy, we generated an overuse‐induced tendinopathy mouse model using intensive treadmill running (ITR) to apply repetitive loads on mouse tendons according to previous study,^[^ ^30^ ^]^ We found evident proteoglycan deposition 4 weeks after ITR in the mid‐portion of Achilles tendons while no proteoglycan was noted in the control mice (Figure S2A, Supporting Information). Moreover, a prominent change in tenocyte morphology was noted (from spindle‐like nuclei to rounded nuclei) (Figure S2A, Supporting Information). CD68^+^ cells were seldom seen in either ITR and control mice, suggesting inflammation did not play a major role in ITR induced tendinopathy (Figure S2B,C, Supporting Information). The changes in TGF‐*β*1 signaling (Active TGF‐*β* concentration and pSmad2 staining) and expression of MMP13 in the ITR model were similar to those observed in the DI model (Figure S2, D–H, Supporting Information). Collectively, these results suggest that altered mechanical force either overloading or overuse induces Achilles tendinopathy, resembling human tendinopathy with high levels of active TGF‐*β*1.

### 2.3 Degeneration in Tendinopathic Tendon Resulted in Inferior Mechanical Property

During the development of tendinopathy in DI mice, tenocytes gradually gained positive terminal deoxynucleotidyl transferase dUTP nick end labeling (TUNEL) signal over time, suggesting a massive apoptosis of tenocytes (Figure S3A,B, Supporting Information). To test the biomechanical properties of Achilles tendons, we performed dynamic mechanical analysis after DI and found significantly less maximum tensile force and stiffness after tendinopathy compared with the normal tendons, suggesting inferior mechanical properties (Figure S3C,D, Supporting Information). Moreover, 8 weeks after the DI (8‐weeks’ DI) was removed, the biomechanical properties of Achilles tendon cannot resolve spontaneously (Figure S3E–G, Supporting Information). Similar to DI tendinopathy mouse model, the expression of TUNEL in the ITR model was significantly increased compared to control mice, suggesting a process of degeneration (Figure S3H,I, Supporting Information). Biomechanical testing of Achilles tendons in ITR mice also showed a significant decrease in maximum tensile force and stiffness compared with the control tendons (Figure S3J,K, Supporting Information). We further compared the mobility of the ITR mice with that of control mice using the CatWalk gait analysis system. Two measures, footprint area and swing time, were used to analyze footprint patterns. ITR mice had a smaller footprint area and longer swing time compared with controls, suggesting an abnormal gait in ITR mice (Figure S3M,N, Supporting Information). Together, these results indicate tendinopathic tendons undergo degeneration leading to inferior mechanical properties.

### 2.4 Transgenic Expression of Active TGF‐*β*1 in Col1 Expression Tissue Induces Tendinopathy

**Figure 3:**
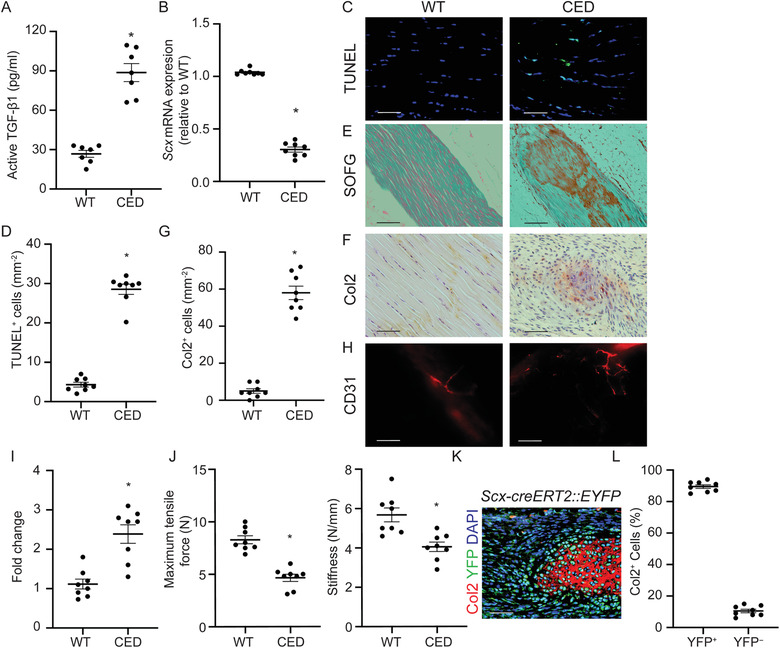
CED mice show an Achilles tendon tendinopathy phenotype. A) Concentration of active TGF‐*β*1 in Achilles tendons of CED and WT mice. B) Message RNA expression of Scx in Achilles tendons of CED and WT mice. C) TUNEL staining and D) quantitative analysis of apoptotic cells in tendons. Scale bar: 50 µm. E) SOFG staining of normal tendon and CED mouse tendons. Proteoglycan (red) and collagenous matrix (green). Scale bar: 200 µm. F) Immunohistochemical staining and G) quantitative analysis of Col2^+^ chondrocytes per tissue area (mm^2^) in Achilles tendons. Scale bar: 100 µm. H) Immunofluorescent staining of CD31^+^ cells and I) quantification of the fold change of blood vessels in CED mice normalized to that of WT mice in the Achilles tendons. Scale bar: 100 µm. J) Quantitative analysis of maximum tensile force and stiffness of Achilles tendons from WT and CED mice. K) Immunostaining of Col2^+^ cells (red) and yellow fluorescent protein (YFP) – positive cells (green) in Scx‐creERT2::EYFP mice 8 weeks after DI. Scale bar: 50 µm. L) Ratio of YFP^+^ and YFP*^−^* cells in Col2^+^ cells in tendinopathic tendon 8 weeks after DI in Scx‐creERT2::EYFP mice. All data are shown as the mean ± standard deviation (*n* = 8 per group). *p < 0.05 compared with WT littermates as determined by unpaired, 2‐tailed Student's *t*‐test.

The human tendinopathy, as well as DI and ITR mouse models, implied that excessively high concentrations of active TGF‐*β*1 in tendon contribute to the pathogenesis of tendinopathy. To further validate the role of elevated active TGF‐*β*1 in tendinopathy, we determined whether high levels of active TGF‐*β*1 could induce spontaneous tendinopathy. In CED, TGF‐*β*1 is constitutively active upon secretion due to a point mutation in the TGFB1 gene.^[^ ^23^, ^26^ ^]^ Previously, we generated a CED mouse model with a CED‐derived TGFB1 mutation (H222D), in which active TGF‐*β*1 is overexpressed in Col1‐enriched tissues,^[^ ^24a–c^ ^]^ such as tendons (**Figure** **3**A). We, therefore, utilized our CED mouse model to determine whether high concentrations of active TGF‐*β* alone are sufficient to initiate spontaneous tendinopathy in Achilles tendon. We first examined the changes in Scx expression in the Achilles tendons of the CED mice because loss of its expression suggests the degeneration of tenocytes.^[^ ^31^ ^]^ We found that mRNA levels of Scx were significantly decreased in CED mice compared with wild‐type (WT) littermates (Figure 3B). TUNEL staining confirmed that tenocytes in CED mice underwent apoptosis (Figure 3C,D). SOFG staining showed disorganized arrangement of ECM in the Achilles tendons of CED mice compared with their WT littermates (Figure 3F). Evident proteoglycan deposition was seen in the Achilles tendon in CED mice at 3 months of age (Figure 3E). The area positive for Col2 staining was significantly greater in Achilles tendons of CED mice compared with WT littermates, suggesting a process of chondrogenesis (Figure 3F,G). Moreover, abundant blood vessels were seen in tendons in CED mice compared with WT controls (Figure 3H,I). Biomechanical testing of Achilles tendons in CED mice showed a significant decrease in maximum tensile force and stiffness compared with the tendons in WT controls (Figure 3J). Altogether, CED mice had a tendinopathy phenotype similar to that of DI and ITR mouse models, suggesting that high concentrations of active TGF‐*β*1 contribute to Achilles tendinopathy. To further investigate the function of active TGF‐*β*1 in tendinopathy, we generated Scx‐creERT2::R26R‐EYFP mice to trace Scx^+^ cells during tendinopathy progression for 8 weeks after DI. Approximately 90% of chondrocytes were derived from the Scx lineage cells (Figure 3K,L). These results suggest that Scx lineage cells gave rise to chondrocytes during tendinopathy in response to excessive active TGF‐*β*1 levels.

### 2.5 Inhibition of Excess Active TGF‐*β*1 Attenuates Tendon Degeneration in DI Mice

**Figure 4:**
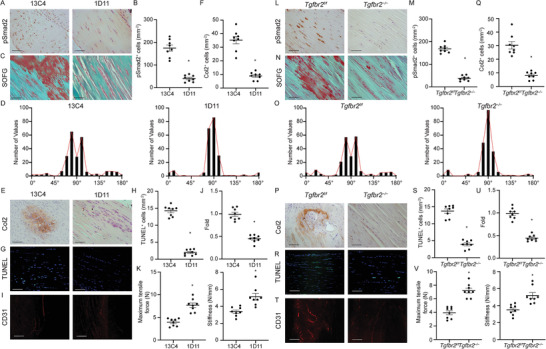
Inhibition of TGF‐*β* signaling attenuates tendinopathy. A–K) TGF‐*β* neutralizing antibody attenuates tendinopathy. Mice are treated with 5 mg kg^−1^ body weight of the TGF‐*β* neutralizing antibody 1D11 or control antibody 13C4 once per week for 8 weeks. A) Immunohistochemical staining and B) quantitative analysis of pSmad2^+^ cells per tissue area (mm^2^) in Achilles tendons. Scale bar: 50 µm. C) SOFG staining of tendons in 13C4‐ or 1D11‐treated mice. Proteoglycan (red) and collagenous matrix (green). Scale bar: 50 µm. D) Frequency distribution of Achilles tendon orientation degree in 13C4‐ or 1D11‐treated mice. E) Immunohistochemical staining and F) quantitative analysis of Col2^+^ chondrocytes per tissue area (mm^2^) in Achilles tendons. Scale bar: 100 µm. G) TUNEL staining and H) quantitative analysis of apoptotic cells in tendons. Scale bar: 100 µm. I) Immunofluorescent staining of CD31^+^ cells and J) quantification of the fold change of blood vessels in 1D11‐treated mice normalized to that of 13C4‐treated mice in the Achilles tendons. Scale bar: 100 µm. K) Quantitative analysis of maximum tensile force and stiffness of Achilles tendons measured 8 weeks after DI operation with 13C4 or 1D11 treatment. All data are shown as the mean ± standard deviation (*n* = 8 mice per group). *p < 0.05 compared with 13C4 treated mice as determined by unpaired, 2‐tailed Student's *t*‐test. L–V) Inducible knockout of Tgfbr2 in Scx^+^ cells attenuates tendinopathy in mice. L) Immunohistochemical staining and M) quantitative analysis of pSmad2^+^ cells per tissue area (mm^2^) in Achilles tendons. Scale bar: 50 µm. N) SOFG staining of tendons in Scx‐creERT2::Tgfbr2*^f/f^* mice or its Tgfbr2*^f/f^* littermates 8 weeks after DI. Proteoglycan (red) and collagenous matrix (green). Scale bar: 50 µm. O) Frequency distribution of Achilles tendon orientation degree in Scx‐creERT2::Tgfbr2*^f/f^* mice or its wildtype controls. P) Immunohistochemical staining and Q) quantitative analysis of Col2^+^ chondrocytes per tissue area (mm^2^) in Achilles tendons. Scale bar: 100 µm. R) TUNEL staining and S) quantitative analysis of apoptotic cells in Achilles tendons. Scale bar: 100 µm. T) Immunofluorescent staining of CD31^+^ cells and V) quantification of the fold change of blood vessels in Scx‐creERT2::Tgfbr2*^f/f^* mice normalized to that of Tgfbr2*^f/f^* mice in the Achilles tendons. Scale bar: 100 µm. T) Quantitative analysis of maximum tensile force and stiffness of Achilles tendons of Scx‐creERT2::Tgfbr2*^f/f^* mice and their Tgfbr2*^f/f^* littermates 8 weeks after DI. All data are shown as the mean ± standard deviation (*n* = 8 mice per group). *p < 0.05 compared with Tgfbr2*^f/f^* mice as determined by unpaired, 2‐tailed Student's *t*‐test.

As DI, ITR, and CED mice showed similar pathological changes, we utilized the DI model for the following experiments. We investigated the effects of TGF‐*β* inhibition on tendinopathy pathogenesis. First, we tested different doses of TGF‐*β*‐neutralizing antibody (1D11) in experimental mice to identify the optimal dose and injection frequency, which was 5 mg per kg of body weight, injected weekly. 1D11 significantly decreased the number of pSmad2^+^ cells in the Achilles tendon compared with vehicle antibody (13C4)^[^ ^23b^ ^]^‐treated DI mice (**Figure** **4**A,B). SOFG staining revealed that the alignment of tendon fibers improved after 1D11 treatment compared with 13C4 (Figure 4C,D). The deposition of proteoglycan was also significantly decreased (Figure 4C). Furthermore, immunostaining analysis of Col2 showed reduced cartilage formation in DI mice after 1D11 treatment, demonstrating the prevention of chondrogenesis (Figure 4E,F). The increased number of TUNEL^+^ apoptotic tenocytes was reduced by 1D11 but not 13C4 (Figure 4G,H). The number of CD31^+^ vessels was increased in the tendons of vehicle‐treated DI mice, and this effect was also attenuated by 1D11 treatment (Figure 4I,J). Moreover, the decreases in the maximum tensile force and stiffness caused by DI were restored after 1D11 treatment (Figure 4K). We further injected 1D11 in the DI‐induced tendinopathy mice from 1 week, 4 weeks, or 8 weeks after the day DI was applied for 8 weeks. The mice were euthanized 12 weeks after 8‐weeks’ DI application (Figure S4A, Supporting Information). Tendinopathy was significantly mitigated with injection of 1D11 antibody from week 1 and week 4 compared to 13C4 injected group. However, injection of 1D11 antibody from week 8 post‐DI, when active TGF‐*β*1 was restored to base line (Figure 2L), did not significantly attenuate tendinopathy measured by mechanical properties, suggesting 1D11 only works when active TGF‐*β*1 is present in Achilles tendons (Figure S4B, Supporting Information). Collectively, these results indicate that inhibition of TGF‐*β* signaling prevents increases in degenerative and apoptotic tenocytes during tendinopathy development, which contributes to disorganized matrix and inferior mechanical properties.

### 2.6 Conditional Knockout of Tgfbr2 in Tenocytes Attenuates Tendinopathy in Mice

To determine whether changes in the tendon were the sequelae of elevated levels of TGF‐*β* signaling in tenocytes, we generated Scx‐creERT2::Tgfbr2*^f^* ^/^ *^f^* mice to specifically delete Tgfbr2 (Tgfbr2*^−/−^*) in the tenocytes upon tamoxifen injection. Deletion of Tgfbr2 in Scx^+^ cells effectively blocked TGF‐*β* signaling cascade in tendons (Figure 4L,M), while the active TGF‐*β*1 concentration did not change (Figure S5, Supporting Information). The deposition of proteoglycan was evident in WT DI mice but not in Tgfbr2*^−/−^* mice (Figure 4N). The matrix arrangement of the Achilles tendon was also maintained in knockout mice (Figure 4O). Col2 production was significantly decreased in DI Tgfbr2*^−/−^* mice compared with WT controls, suggesting an attenuated chondrogenesis in DI Tgfbr2*^−/−^* mice (Figure 4P, Q). TUNEL staining revealed that deletion of Tgbfr2 in tenocytes prevented tenocyte apoptosis after DI compared with WT controls (Figure 4R,S). The number of blood vessels in DI Tgfbr2*^−/−^* mice was also significantly lower than that in WT mice (Figure 4T,U). Mechanical analysis revealed that the decreased maximum tensile force and stiffness was significantly attenuated in Tgfbr2*^−/−^* mice compared with WT littermates, indicating restored mechanical properties (Figure 4V). These data confirm that active TGF‐*β* signaling in tenocytes contributes to the pathogenesis of tendinopathy.

### 2.7 Integrin *α*v*β*6 Induces TGF‐*β* Activation in Response to Mechanical Load in Tendons

**Figure 5:**
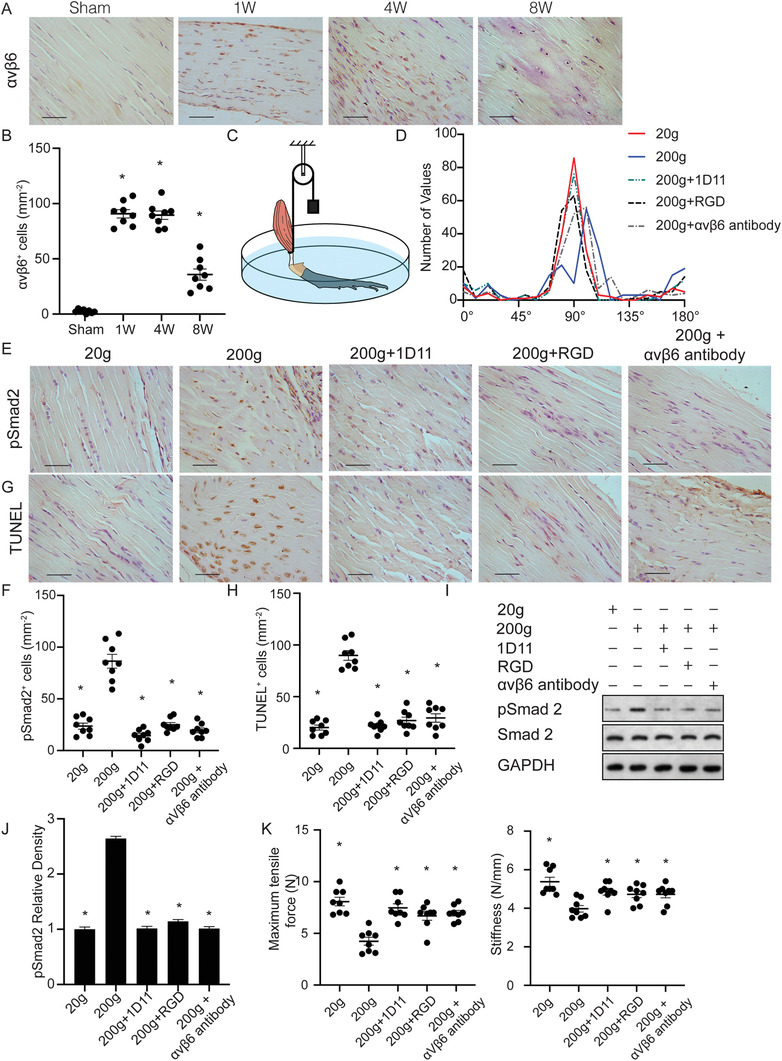
Integrin *α*v*β*6 active latent TGF‐*β* in response to mechanical stress drives the progression of tendinopathy. A) Representative images and B) quantification of immunostaining of tendon sections with antibodies against *α*v*β*6 at 1, 4, and 8 weeks after DI or sham processing. Scale bar: 50 µm. All data are shown as the mean ± standard deviation (*n* = 8 mice per group). *p < 0.05 compared with sham group as determined by one‐way analysis of variance. C) A schematic diagram of the ex vivo tensile model. D) Frequency distribution of tendon fiber orientation. E,G) Representative images and F,H) quantification of E,F) pSmad2^+^ cells and (G, H) TUNEL^+^ cells. Scale bars: 50 µm. I) Western blot of pSmad2 and Smad2 levels in tendon lysates. J) Quantification of pSmad2 relative density relative to physiological loading group (20 g) in tendon lysates after above‐mentioned treatment. K) Quantitative analysis of maximum tensile force and stiffness of tendons. All data from B, F, H, J, and K are shown as the mean ± standard deviation (*n* = 8 per group). *p < 0.05 compared with 200 g loaded group as determined by one‐way analysis of variance.

To understand the mechanism of TGF‐*β* activation in tendons during tendinopathy, we first stained Achilles tendon sections with integrin *α*v*β*6 antibody since the *α*v*β*6 integrins are known to mediate cell‐induced conformational change of TGF‐*β* latent complex to release active TGF‐*β*. The expression of integrin *α*v*β*6 was consistent and continuing increase in the midportion of Achilles tendon from 1 to 8 weeks after DI was applied compared with the sham control (**Figure** **5**A,B). The pattern of elevation of *α*v*β*6 expression was similar to that of pSmad2^+^ cells. To determine a causal relationship, we generated an ex vivo Achilles tendon stretch‐loading model (Figure 5C). 1D11, RGD peptide, *α*v*β*6 antibody, and vehicle were applied to culture medium. We found active TGF‐*β*1 concentration was significantly increased in overloaded (200 g) tendons compared to physiological loading (20 g), while 1D11, RGD, or *α*v*β*6 antibody treatment significantly decreased active TGF‐*β*1 levels in overloaded tendons (Figure S6A, Supporting Information). There were no statistical significances of active TGF‐*β*1 concentrations among culture medium, suggesting few active TGF‐*β*1 were released to culture medium (Figure S6B, Supporting Information). The tendon fiber distribution was disrupted by overloading within 24 h, while 1D11, RGD and *α*v*β*6 antibody treatment improved the fiber alignment (Figure 5D). Immunostaining revealed that overloading significantly increased the number of pSmad2^+^ cells compared with physiological loading (Figure 5D, E). TUNEL^+^ tenocytes were also significantly increased, suggesting apoptosis of tenocytes after overloading (Figure 5F, G). In contrast, 1D11, RGD peptide, and *α*v*β*6 antibody inhibited overload‐induced phosphorylation of Smad2 and apoptosis in tendon cells (Figure 5F, G). This result was confirmed by western blot analysis (Figure 5H, I). Moreover, the biomechanical properties, including maximum tensile force and stiffness, were decreased after overloading but restored in the 1D11, RGD, and *α*v*β*6 antibody groups (Figure 5J). These results show that integrin *α*v*β*6 induces TGF‐*β*1 activation in response to mechanical force to regulate tenocyte function.

### 2.8 Conditional Knockout of Integrin *α*v in Tendon Prohibits Tendinopathy in Tendinopathy Mice

**Figure 6:**
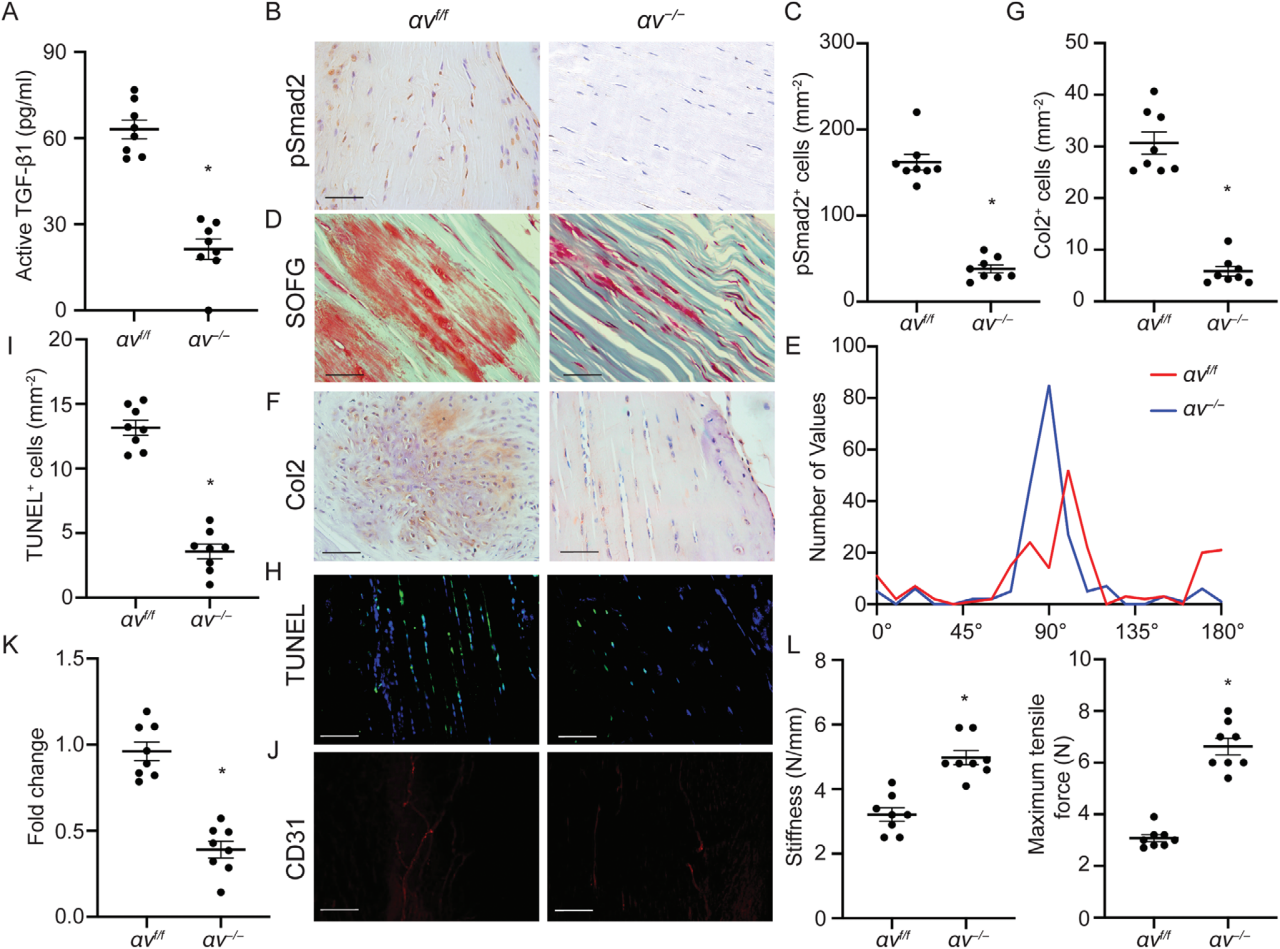
Inducible knockout of *αv* in Scx^+^ cells attenuates tendinopathy. A) Active TGF‐*β*1 concentration of tendon sections from Scx‐creERT2::*αv^f/f^* and their WT *αv^f/f^* littermates upon tamoxifen injection. B) Immunostaining and C) quantification of pSmad2^+^ cells in tendons. Scale bar: 50 µm. D) SOFG staining of tendons in WT or Scx‐creERT2::*αv^f/f^* mice. Proteoglycan (red) and collagenous matrix (green). Scale bar: 50 µm. E) Frequency distribution of tendon fiber orientation. F) Immunohistochemical staining and G) quantitative analysis of Col2^+^ chondrocytes per tissue area (mm^2^) in Achilles tendons. Scale bar: 100 µm. H) TUNEL staining and I) quantitative analysis of apoptotic cells in tendons. Scale bar: 100 µm. J) Immunofluorescent staining of CD31^+^ cells and K) quantification of the fold change of blood vessels in Scx‐creERT2::*αv^f/f^* mice normalized to that of WT mice in the Achilles tendons. Scale bar: 100 µm. Quantitative analysis of L) maximum tensile force and stiffness of tendons. All data are shown as the mean ± standard deviation (*n* = 8 mice per group). *p < 0.05 compared to *αv^f/f^* mice as determined by unpaired, 2‐tailed Student's *t*‐test.

To determine the role of integrin αv in activation of TGF‐*β* in tendinopathy, we generated Scx‐creERT2::*αv^f/f^* (*αv* ^−/−^) mice to delete integrin *α*v expression in tenocytes upon tamoxifen injection (Figure S7A, Supporting Information). Immunostaining of Achilles tendon sections validated that *α*v*β*6 expression was diminished in tenocytes in DI *αv^−/−^* mice (Figure S7B, Supporting Information). To evaluate whether deletion of *α*v was sufficient to prevent mechanically induced tendinopathy, we further subjected *αv^−/−^* mice to DI for 4 weeks or sham treatment. As expected, the concentration of active TGF‐*β*1 in *αv^−/−^* mice was significantly lower than that in WT littermates (**Figure** **6**A). Phosphorylated Smad2^+^ tenocytes were significantly reduced in *αv^−/−^* mice compared with WT littermates (Figure 6B, C). The *αv^−/−^* mice showed attenuated tendon ECM and morphologic alteration by SOFG staining and alignment quantification (Figure 6D, E). The deposition of proteoglycan and amount of Col2 were reduced in *αv^−/−^* mice compared with WT controls (Figure 6D–F). Massive tenocyte apoptosis was observed in the WT mice, whereas the knockout of *α*v significantly reduced TUNEL^+^ cells (Figure 6G,H). Similarly, CD31^+^ blood vessels were also decreased (Figure 6I, J). Statistically significant increases in maximum tensile force and stiffness were seen in *αv^−/−^* mice compared with WT littermates, indicating restored mechanical properties (Figure 6K). Taken together, conditional knockout of integrin *αv* impedes tendinopathy in mice.

## 3 Discussion

Major progress has been made in understanding the involvement of inflammatory factors in tendinopathy. However, acute inflammatory cells have never been confirmed in chronic tendinopathy. Thus, classic treatments based on analgesic and nonsteroidal anti‐inflammatory drugs and passive physical therapy are often insufficient. Tendons are constantly subjected to mechanical loads in vivo, which influence the function and structure of musculoskeletal tissues. Therefore, defining the role of mechanical loading in tendons is crucial to understanding the pathophysiology of tendinopathy. In the present study, we used in vivo and ex vivo models to show that mechanical overloading or overuse induces TGF‐*β* activation via integrin *α*v*β*6 in tendon, leading to degeneration, apoptosis, and chondrogenesis. Excessive active TGF‐*β* also results in excessive blood vessel formation in the Achilles tendons of mice.

Excessive or repetitive mechanical loading is the most common cause of tendinopathy.^[^ ^28^ ^]^ Our study and previous studies have found that tenocytes and TSPCs are the effector cells in response to mechanical loading.^[^ ^6^, ^32^ ^]^ In normal physiological situations, appropriate mechanical loading produced by body weight and muscle force is necessary to activate TGF‐*β*, which modulates several essential cellular processes in tissue development, differentiation, and homeostasis. TGF‐*β* signaling also plays a major role in the genesis of tendon and promotes maintenance and recruitment of tendon progenitors.^[^ ^33^ ^]^ Properly activated TGF‐*β* modulates anabolic activity of tenocytes and maintains tendon homeostasis. However, in pathological conditions, as we found in the present study, overloaded or repetitive tensile stresses activate excessive TGF‐*β* and result in functional transition of tenocytes, leading to pathological changes in tendon.

We found excessive activation of TGF‐*β* together with hypercellularity in the tendon at the onset of tendinopathy. These findings indicate that more tenocytes are generated for the repair and/or remodeling of tendons by active TGF‐*β* in response to mechanical loading on the tendons. However, the poor regenerative capacity of tendon results in inferior tissue quality, and the endogenous tenocytes/TSPCs thus fail to functionally restore damaged tissue. Indeed, we found that excessive release of active TGF‐*β* leads to massive degeneration and apoptosis of tenocytes/TSPCs in the tendon proper as well as chondrogenic lesions during the progression of tendinopathy, consistent with previous findings.^[^ ^6a^ ^]^ This may be either through a Smad‐dependent mechanism^[^ ^34^ ^]^ or a JNK pathway.^[^ ^35^ ^]^

Using Scx as the promoter, we performed lineage tracing and found that the number of Scx^+^ cells increased during the progression of tendinopathy, suggesting that the resident cells, either local TSPCs or tenocytes, are involved. Previous studies have shown increased chondrocytes in degenerative tendons.^[^ ^36^ ^]^ Our results confirmed that Scx^+^ cells contribute to tendon degeneration by giving rise to the chondrocyte lineage, which, at later stages, manifests as increased amounts of proteoglycans and Col2. We further showed that the role of mechanical loading in chondrogenesis is dependent on TGF‐*β*‐mediated pathways. Furthermore, in this study, we found that inhibition of TGF‐*β* signaling by neutralizing antibody or knockout of Tgfbr2 in Scx^+^ cells mitigated cartilage formation in tendinopathic tendons. Therefore, TGF‐*β* plays a pivotal role in differentiating Scx^+^ cells to chondrocytes.

Given that healthy tendons typically are poorly vascularized, neovascularization after tendon injury is associated with degeneration rather than functional repair. Indeed, we found that vessel formed within tendon in response to mechanical loading. Vasculature gradually increased during the development of tendinopathy, and the newly formed blood vessels were also derived from Scx lineage cells. Importantly, blockage of TGF‐*β* signaling significantly mitigated angiogenesis. Although local Scx^+^ cells are the primary cause of both chondrogenesis and angiogenesis, it is also plausible that circulating progenitor cells may be recruited from the periphery by the excessive activated TGF‐*β*.

Using a static tensile load with calibrated stainless‐steel weights (20 g for physiological loads and 200 g for pathological loads) to Achilles tendon, we have demonstrated that integrin *α*v*β*6 induces TGF‐*β*1 activation in response to mechanical force to regulate tenocyte function. However, we noticed that overload induced slight changes in cell distribution. This change may be due to the disruption of collagen fibers after over loading (Figure 5D). However, the cellular changes are not as rapid in tendinopathy patients. Additionally, it would be more accurate to apply loading individually based on the weight of each mouse. Thus, the ex vivo model could not mimic all aspects of the human conditions. We thus utilized *αv^−/−^* mice to delineate the role of integrin *α*v*β*6 in the development of tendinopathy because blockage of *α*v will eliminate the expression of *α*v*β*6 since *β*6 can only partner with *α*v^[^ ^37^ ^]^ and no specific *α*v*β*6 knockout mice are available. Our results imply that the integrin‐RGD associate may result in a tensile force‐dependent conformational change of latent complex to release active TGF‐*β*. Indeed, this force‐dependent mechanism is supported by experiments that demonstrated both external stretching or increasing intracellular tension directly activate latent TGF‐*β*1 from the latent TGF‐*β* binding protein‐1 (LTBP‐1) in the extra cellular matrix.^[^ ^26^, ^38^ ^]^ Although involvement of TGF‐*β* signaling in pathogenesis of tendinopathy has been reported, we are the first to demonstrate that integrin *α*v*β*6 activation of excessive TGF‐*β*1 is the molecular mechanism of tendinopathy.

In the present study, we have demonstrated that active TGF‐*β*1 levels were significantly increased during the pathogenesis of the energy‐storing tendon Achilles in human and mice. However, such data do not necessarily mean that tendinopathy of positional tendons such as rotator cuff will share the same mechanisms. Additionally, it is possible that *α*v‐independent mechanism is involved in TGF‐*β*1 activation. But this concept may be more evident in the diseases where mechanical forces were not involved.

In conclusion, we found that the activation of TGF‐*β* by integrin *α*v*β*6 is central to mechanical signal transduction within tendon. The pathological mechanical loading on the tendon drives aberrant overactivation of TGF‐*β* through integrin *α*v*β*6. Blockage of *α*v*β*6 using antibody or in *αv* knockout mice significantly reduced mechanical force–induced high TGF‐*β* signaling, leading to attenuated tendon degeneration, chondrogenesis, and blood vessel formation in tendon. Thus, *α*v*β*6 activation of TGF‐*β* drives tendinopathy development. The current results not only provide the pathomechanism of tendinopathy but can also guide further preclinical and clinical studies to treat tendinopathy in humans.

## 4 Experimental Section

### Human Subjects

This study was approved by the internal review board of Shanghai Sixth People's Hospital (Shanghai, China, 2021‐KY‐78(K)). The Achilles tendons from 8 patients with symptomatic Achilles tendinopathy were recruited from a sports medicine clinic (5 men and 3 women; age range, 42–61 years). The presence of Achilles tendinopathy was identified by ultrasound. All patients completed the VISA‐A scoring system to evaluate the severity of Achilles tendinopathy.^[^ ^39^ ^]^ All patients failed the eccentric training program (3‐month) and received high volume injection (HVI) of 10 mL 0.5% bupivicaine and 30 mL saline into the pre‐Achilles space. Achilles tendon biopsies were collected through percutaneous ultrasound‐guided biopsy under local anesthesia at the tendinopathic site (mid‐potion of Achilles tendon) using a 14G trucut biopsy needle before HVI.^[^ ^40^ ^]^ Exclusion restrictions will be applied to comorbidities (diabetes, heart disease etc.), previous intratendinous corticosteroid/platelet‐rich plasma/stem cell injection, extracorporeal shockwave therapy or systemic steroid or methotrexate treatments. Healthy hamstring (semitendinosis) tendons collected from 8 patients (6 men and 2 women; age range, 19–52 years) undergoing surgical anterior cruciate ligament reconstruction were used as controls.

### Mice

The protocols of this study were carried out with the approval of the Animal Care and Use Committee of The Johns Hopkins University and the internal review board of the Shanghai Sixth People's Hospital. All mice were maintained in the animal facility of The Johns Hopkins University School of Medicine. C57BL/6J (WT) mice were purchased from the Jackson Laboratory (Bar Harbor, ME). DI was performed on the right foot of each mouse to generate an overloading‐induced tendinopathy mouse model, adjusted as in a previously described procedure.^[^ ^23b^ ^]^ To make an immobilization device, TempAssure 0.5‐mL PCR tubes (USA Scientific Inc, Ocala, FL, catalog no. 1405‐8100) were used. The bottoms (15 mm from the bottom) and the caps were first removed from the tubes. Two holes were then drilled 4 mm from the top of the tubes on opposite sides (180° apart). Three‐month‐old mice were anesthetized by inhalation of isoflurane. After anesthetization, the right ankle of the mouse was inserted into the top of the tube in dorsiflexion. A 2‐cm iron wire was inserted through the 2 holes and above the ventral part of the ankle to secure the device in place. When this device was applied, the calf muscles of the mice were tight. Therefore, dorsiflexion of the ankle joint was restricted, and the foot was bent into a talipes calcaneus position. The devices were applied to mice for 12 h every day for 1, 4, or 8 weeks as described in the Results. The mice were allowed to move freely in the cages. At the end of each experimental time point, the devices were discarded, and mice were humanely sacrificed. Non‐immobilized littermates were used as controls.

For ITR,^[^ ^30^ ^]^ briefly, 4‐month‐old C57BL/6 mice received training for treadmill running (Columbus Instruments, Columbus, OH) at 13 m min^−1^, 15 min day^−1^, and 5 days week^−1^ in the first week. The mice then run at 13 m min^−1^ for 3 h day^−1^, 4 h day^−1^, and 5 h day^−1^ for 5 days in the second, third, and fourth weeks, respectively. The mice in control group received the first week's treadmill training but no treadmill running in the following weeks and were allowed to move freely in cages. The mice will be sacrificed after the last treadmill running at week 4, and their Achilles tendons were harvested and subsequently analyzed.

The 8‐week‐old Tgfbr2*^f/f^* (stock no. 012603) and R26R‐EYFP (stock no. 006148) mouse strains were purchased from the Jackson Laboratory. Scx‐creERT2 mice were provided by Dr. Ronen Schweitzer at Shriners Hospital for Children (Portland, OR). The integrin *αv^f/f^* mouse strain was obtained from the laboratory of Dr. Andrew Burich, Benaroya Research Institute at Virginia Mason (Seattle, WA).

CED mice were generated in the laboratory as previously described, in which the CED‐derived TGF‐*β*1 mutation (H222D) was specifically expressed by cells driven by a 2.3‐kb Col1*α*1 promoter. 3‐month‐old male CED mice was used for analyzing spontaneous tendinopathy and 3‐month‐old male WT mice as controls.

Scx‐creERT2::R26R‐EYFP mice were generated by crossing Scx‐creERT2 mice with R26R‐EYFP mice. The authors treated 3‐month‐old Scx‐creERT2::R26R‐EYFP male mice with 80 mg kg^−1^ body weight of tamoxifen and performed the DI procedure immediately after injection. Mice were continued to be treated every 2 weeks for 8 weeks and humanely euthanized 8 weeks after injection.

For the antibody treatment experiments, 3‐month‐old DI‐processed male mice were intraperitoneally injected with TGF‐*β* neutralizing antibody 1D11^[^ ^41^ ^]^ (R&D Systems, Minneapolis, MN) at 5 mg kg^−1^ body weight 3 times per week or with 13C4 (R&D Systems) from the day of DI or from 1 week, 4 weeks and 8 weeks after DI for 4 weeks. The mice were humanely euthanized 12 weeks after DI.

Scx‐creERT2::Tgfbr2*^f/f^* mice were generated by crossing Scx‐creERT2 mice with Tgfbr2*^f/f^* mice. 3‐month‐old Scx‐creERT2::Tgfbr2*^f/f^* mice were injected with tamoxifen (80 mg kg^−1^, intraperitoneal injection) or vehicle and underwent DI on the right ankle 3 days after injection. The mice were treated with either tamoxifen or vehicle once a week for 8 weeks and humanely euthanized the mice 8 weeks after DI.

Scx‐creERT2::*αv^f/f^* mice were generated by crossing Scx‐creERT2 mice with *αv^f/f^* mice. Three‐month‐old Scx‐creERT2::Tgfbr2*^f/f^* mice were injected with tamoxifen (80 mg kg^−1^, intraperitoneal injection) or vehicle. Three days after injection, mice underwent DI and were injected intraperitoneally with either 80 mg kg^−1^ body weight of tamoxifen or vehicle once a week until the mice were humanely euthanized 8 weeks after DI.

### Specimen Collection

Mice were euthanized with inhalation of carbon dioxide and perfusion‐fixed with 4% paraformaldehyde through the left ventricle for 5 min. The Achilles tendons were dissected and fixed in 4% paraformaldehyde for 24 h, and then embedded in paraffin, optimal cutting temperature compound (VWR International, Radnor, PA, catalog no. 25608‐930), or matrix containing 8% gelatin (Sigma‐Aldrich St. Louis, MO, catalog no. G1890), 20% sucrose (Sigma‐Aldrich, catalog no. S9378), and 2% polyvinylpyrrolidone (Sigma‐Aldrich, catalog no. PVP40) at −80 °C as adjusted from a previous protocol.^[^ ^42^ ^]^ Most analyses were of paraffin‐embedded specimens, whereas detection of fluorescent staining of Col2, GFP, and TUNEL was performed in optimal cutting temperature compound–embedded frozen specimens. The visualization of CD31 was clearer in gelatin matrix–embedded frozen samples.

### Histochemistry, Immunohistochemistry, and Immunofluorescence Analysis

Four‐µm and 40‐µm sagittal sections were created using a paraffin microtome (for paraffin blocks) and a Microm cryostat (Thermo Fisher Scientific, Waltham, MA) (for frozen blocks). 4‐µm‐thick sections of tendon were processed for HE staining and Safranin‐O (Sigma‐Aldrich, catalog no. S2255) and fast green (Sigma‐Aldrich, catalog no. F7252) staining. Sections were processed for TUNEL staining according to the manufacturer's protocol (Promega, Madison, WI, catalog no. G3250, for Figures 2, 3, 4F,P, and 6G; Promega, catalog no. G7360, for Figure 5F). Immunohistochemistry and immunofluorescence staining of human and mouse sections was performed using a standard protocol. Both dewaxed paraffin sections and frozen sections were heated to 99 °C for 20 min in Target Retrieval Solution (Dako, Santa Clara, CA, S1699, 1:10) for antigen retrieval. Sections were incubated with primary antibodies to human CD68 (Abcam, Cambridge, UK, ab955, 1:200), human Col2 (Abcam, ab34712, 1:100), human MMP13 (Abcam, ab3208, 1:40), human/mouse pSmad2 (Santa Cruz Biotechnology, Dallas, TX, sc‐11769, 1:50), mouse Col2 (Abcam, ab185430, 1:100), mouse CD68 (Abcam, ab125212, 1:100), mouse MMP13 (Abcam, ab219620, 1:100), mouse green fluorescent protein (Abcam, ab290, 1:200), and integrin *α*v*β*6 (Bioss Antibodies, Woburn, MA, bs‐5791R‐Biotin, 1:100) overnight at 4 °C. For immunohistochemical staining, secondary antibodies were incubated in blocking solution for 1 h at room temperature, and chromogenic substrates (Dako, Agilent Technologies, Santa Clara, CA, K3468) were used to detect the immunoactivity followed by counterstaining with hematoxylin (Sigma‐Aldrich, H9627). For immunofluorescence staining, secondary antibodies conjugated with fluorescence were used. Isotype‐matched controls, such as polyclonal rabbit IgG (R&D Systems, Minneapolis, MN, AB‐105‐C), polyclonal goat IgG (R&D Systems, AB‐108‐C), and monoclonal rat IgG2A (R&D Systems, 54447) under the same concentrations and conditions were used as negative controls. An Olympus DP71 microscope (Olympus, Waltham, MA) was used for imaging samples.

The numbers of positively stained cells in 5 random visual fields per stain in 5 sequential sections in each group were counted and normalized to the number per square millimeter in the area of interest. Quantifications were performed using ImageJ, version 1.48u4, software (National Institutes of Health, Bethesda, MD).

### Tendon Fiber Alignment Analysis

The SOFG pictures were used to analyze fiber alignment. The pictures were changed to 8‐bit graph followed by orientation analysis using an OrientationJ plugin of ImageJ^[^ ^43^ ^]^ with the following parameters, Local window *δ*: 13 pixels; Gradient: Cubic Spline, Vector Field: Grid size: 50, Length vector: Maximum, Scale vector (%): 94. The orientation was evaluated for every patch of the image‐based which allowed to visualize the orientation of the vector. Frequency distribution of orientation degree was analyzed using Prism9.

### Total and Active TGF‐*β*1 Analysis

The Achilles tendon samples were cleaned of surrounding adipose tissues, rinsed with Phosphate‐Buffered Saline (PBS, 40 µL per mouse), and cut into 0.5–1 mm pieces in PBS. An equal volume of RIPA buffer (89900, Thermo Fisher Scientific) containing protease inhibitors (78442, Thermo Fisher Scientific) was added. The tendon tissues were lysed on ice for 30 min with gentle agitation followed by centrifugation to remove debris. The total protein concentration was quantified using a total protein assay (23225, Thermo Fisher Scientific). To normalize the total protein concentration, the samples were diluted to same concentration which fell within the linear range of the standard curve of the TGF‐*β*1 ELISA Development kits (human: R&D Systems, DB100B; mouse: R&D Systems, MB100B). The concentrations of total and active TGF‐*β*1 were examined immediately after dilution following the manufacturer's instructions and obtained by comparing to the standard curve and shown as mass vs volume (pg mL^−1^). Specifically, for total TGF‐*β*1 measurement, samples were first treated with 1 N HCl (1 µl/50 µl supernatant) for 15 min at room temperature followed by neutralization with an equal volume of 1 N NaOH before analysis by ELISA. For the measurement of active TGF‐*β*1 which was the activated form of TGF‐*β*1 within tendons in human and mice, samples were analyzed by ELISA without acid treatment.

### Real‐Time PCR

Tendon tissues were dissolved in TRIzol Reagent (Invitrogen, Carlsbad, California, USA) to extract total RNA according to the manufacturer's instructions. Total RNA was reverse‐transcribed using SuperScript III First‐Strand Synthesis System (Invitrogen) and applied with SYBR Green PCR Master Mix (Invitrogen). The primers of Scx were 5′‐CTTCACTGCGCTGCGCACACTCATCC‐3′ and 5′‐GCTCTCCGTGACTCTTCAGTGGCATCC‐3′. The primers of integrin *α*v (Itgav) were 5′‐GCCAGACCCGTTGTCACTGTAAATGC‐3′ and 5′‐CGTCGGATGGCTCCCTTCTGCTTGAG‐3′. Real‐time PCR was performed using QuantStudio 3 Real‐Time PCR System (Invitrogen).

### Biomechanical Testing

To evaluate the mechanical properties of tendons, the load to failure and stiffness using a Dynamic Mechanical Analyzer Q800 (TA Instruments, New Castle, DE) was analyzed. The tendons were secured to the force gauges. The gauge length was set at 4 mm, and the samples were tested at a creep rate of 3 mm min^−1^. Maximum tensile force was recorded, and stiffness was calculated at the slope of the linear portion of the stress/strain curve.

### CatWalk Analysis

The CatWalk gait analysis system (Catwalk XT 10.6, Noldus Information Technology, Wageingen, The Netherlands) was used to measure gait parameters of freely moving mice as described previously.^[^ ^44^ ^]^ The CatWalk system comprises a glass plate (length, 110 cm) with a fluorescent tube that beams a light into the entire long edge of the glass walkway. A high‐speed color video camera and recording and analysis software were used to assess the gait of mice. All mice received daily training for 1 week, which contained over 6 times cross‐back walk on glass floor. After training, the mice were placed individually in the CatWalk walkway in a dark room. As the mice walked along the glass walkway floor, the footprint images were captured by the video recorder. CatWalk software, version 7.1, automatically labeled all areas containing pixels above the set threshold (7 pixels). Footprint area (complete surface area contacted by the paw during the stance phase) and swing time (duration in seconds of no paw contact with the glass plate during a step cycle) were analyzed.

### Tendon Ex Vivo Tensile Model

In 3‐month‐old C57BL/6 mice, the feet was removed and the gastrocnemius muscle was mechanically isolated from proximal tissues. Calcanei were anchored to the bottom of culture dishes. Achilles tendons were attached by surgical sutures at the tendon‐muscle junction, allowing the tendon‐suture unit to be mounted in‐line with a weight. Physiological loading (20 g) or overloading (200 g) was applied to Achilles tendons. TGF‐*β* neutralizing antibody 1D11 (5.0 µg mL^−1^, R&D Systems), RGD peptide (2 µmol L^−1^, 3498, Tocris Bioscience, Bristol, UK), integrin *α*v*β*6 antibody (100 µg mL^−1^, ab77906, Abcam), or phosphate‐buffered saline (as a control) was added to Dulbecco's modified Eagle's medium (D5030, Sigma‐Aldrich) supplemented with 1% penicillin‐streptomycin (MT30001CI, Fisher Scientific, Pittsburgh, PA). Achilles tendons were collected 24 h after the treatments.

### Western Blot

Western blot analyses were conducted on the protein lysate from Achilles tendons in the ex vivo tensile models. Briefly, the tendon samples were rinsed with PBS and cut into 0.5–1 mm pieces in PBS. An equal volume of RIPA buffer (89900, Thermo Fisher Scientific) containing protease inhibitors (78442, Thermo Fisher Scientific) was added. The tendon tissues were lysed on ice for 30 min with gentle agitation followed by centrifugation to remove debris. The total protein concentration was evaluated by detergent compatible protein assay (Bio‐Rad Laboratories), separated by SDS‐PAGE (sodium dodecyl sulfate‐polyacrylamide gel electrophoresis), and blotted on polyvinylidene fluoride membranes (Bio‐Rad Laboratories). Specific antibodies recognizing mouse pSmad2 (3101, Cell Signaling Technology, Danvers, MA, 1:1 000), Smad2 (3103, Cell Signaling Technology, 1:1 000), and GAPDH (8884, Cell Signaling Technology, 1:1000) were applied, and the proteins were detected using an enhanced chemiluminescence kit (RNP2108, Amersham Biosciences, Little Chalfont, UK). The original blots were provided in the source data file. Quantitation of Western blot signals was performed with ImageJ software. First, the western blot films were scanned and stored into JPEG format. Load the image into ImageJ, under the “analyze” menu set “Set Measurements. Check the “Grey Mean Value” from the checkboxes. Define the selection as the area of interest and record measurements.

### Statistical Analysis

All statistical analyses were performed using SPSS, version 15.0, software (IBM Corp, Armonk, NY). Data are expressed as mean ± standard deviation. For 2‐group comparisons, statistical analysis was performed using unpaired, 2‐tailed Student's *t* test. For other comparisons, statistical analysis was performed using one‐way analysis of variance, followed by Fisher's least significant differences post‐hoc test to determine significance between groups. Differences were considered significant at *p* < 0.05.

### Study approval

The experimental protocols were reviewed and approved by the Institutional Animal Care and Use Committee of The Johns Hopkins University.

## Conflict of Interest

The authors declare no conflict of interest.

## Author contributions

X.W. and S.L. contributed equally to this work. X.W. generated the ideas and designed experiments. X.W. and S.L. performed the most experiments. T.Y. and S.A. performed ITR analysis. R.D. and X.T. performed mechanical property test. W.Z., D.P., J.C., and M.W. participated in experimentation. A.C. and X.C. provided critical suggestions. X.W. wrote the manuscript.

## Supporting information

Supporting Information

## Data Availability

The data that support the findings of this study are available from the corresponding author upon reasonable request.
