# Supplementary material for: Inhibition of Integrin αvβ6 Activation of TGF‐β Attenuates Tendinopathy
Source: Adv Sci (Weinh). 2022 Feb 15;9(11):2104469. doi: 10.1002/advs.202104469 (PMC9009110; doi:10.1002/advs.202104469)
Supplement: Supplementary file 1 — Supporting Information [file ADVS-9-2104469-s001.pdf]

ADVANCED SCIENCE
Open Access

Supporting Information

for *Adv. Sci.,* DOI: 10.1002/advs.202104469

Inhibition of integrin αvβ6 activation of TGF-β attenuates tendinopathy in mice

*Xiao Wang, Shen Liu, Tao Yu, Senbo An, Ruoxian Deng, Xiaohua Tan, Janet Crane, Dayu Pan, Mei Wan, Andrew Carr, Xu Cao**

Supporting Information

Inhibition of integrin αvβ6 activation of TGF-β attenuates tendinopathy in mice

*Xiao Wang[1], Shen Liu[1], Tao Yu[1], Senbo An[1], Ruoxian Deng[1], Xiaohua Tan[1], Janet Crane[1], Dayu Pan[1], Mei Wan[1], Andrew Carr[2], Xu Cao[1]**

*Corresponding author. Email: xcao11@jhmi.edu

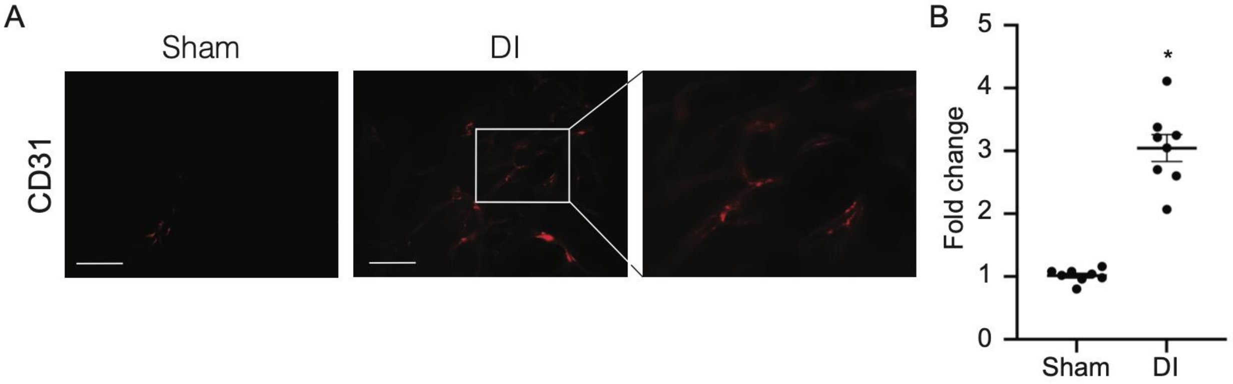

Figure S1. Blood vessels were increased in the tendinopathic tendons.

(A) Immunofluorescent staining of $CD31^+$ cells and (**B**) quantification of the fold change of blood vessels in mice 8 weeks after DI normalized to that of sham mice in the Achilles tendons. Scale bar: 200 µm. All data are shown as the mean ± standard deviation (n = 8 mice per group). *$p < 0.05$ compared with sham group as determined by unpaired, 2-tailed Student's *t*-test.

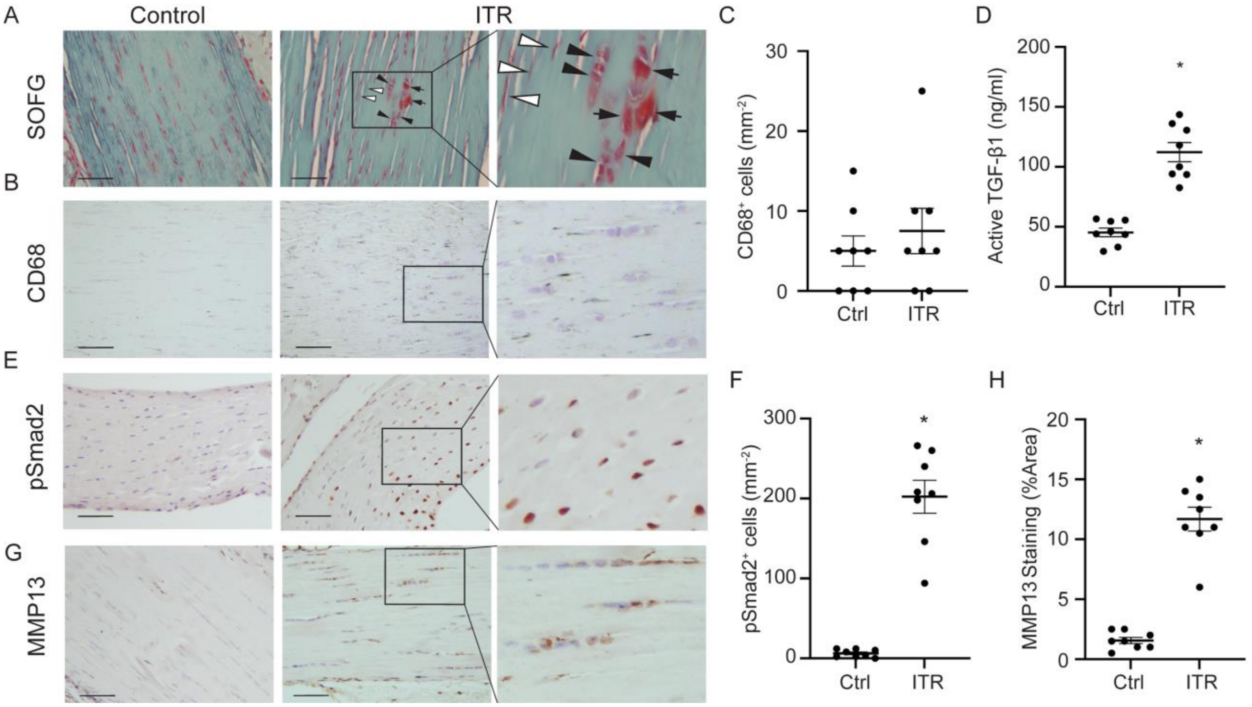

Figure S2. Active TGF-β1 levels were elevated in intensive treadmill running (ITR) induced Achilles tendinopathy in mice.

(**A**) Safranin-O and fast green (SOFG) staining of normal tendon (left column) and tendinopathic tendons (middle and right column) induced by ITR. Black arrows, proteoglycan (red), black arrowheads, chondrocyte-like cells; open arrowheads, normal tenocytes. Graph on the right column is the higher magnification of boxed area in middle column graph. Scale bar: 50 μm. (**B, E, G**) Immunohistochemical staining and (**C, F, H**) quantitative analysis of (**B, C**) $CD68^+$ macrophages, (**E, F**) $pSmad2^+$ cells and (**G, H**) $MMP13^+$ cells per tissue area (mm^2) in Achilles tendons. Black arrowheads, $MMP13^+$ cells. Scale bar: 50 μm. (**D**) The concentration of active TGF-β1 in Achilles tendon in sham and ITR mice. All data are shown as the mean ± standard deviation (n = 8 per group). *$p < 0.05$ as determined by unpaired, 2-tailed Student's *t*-test.

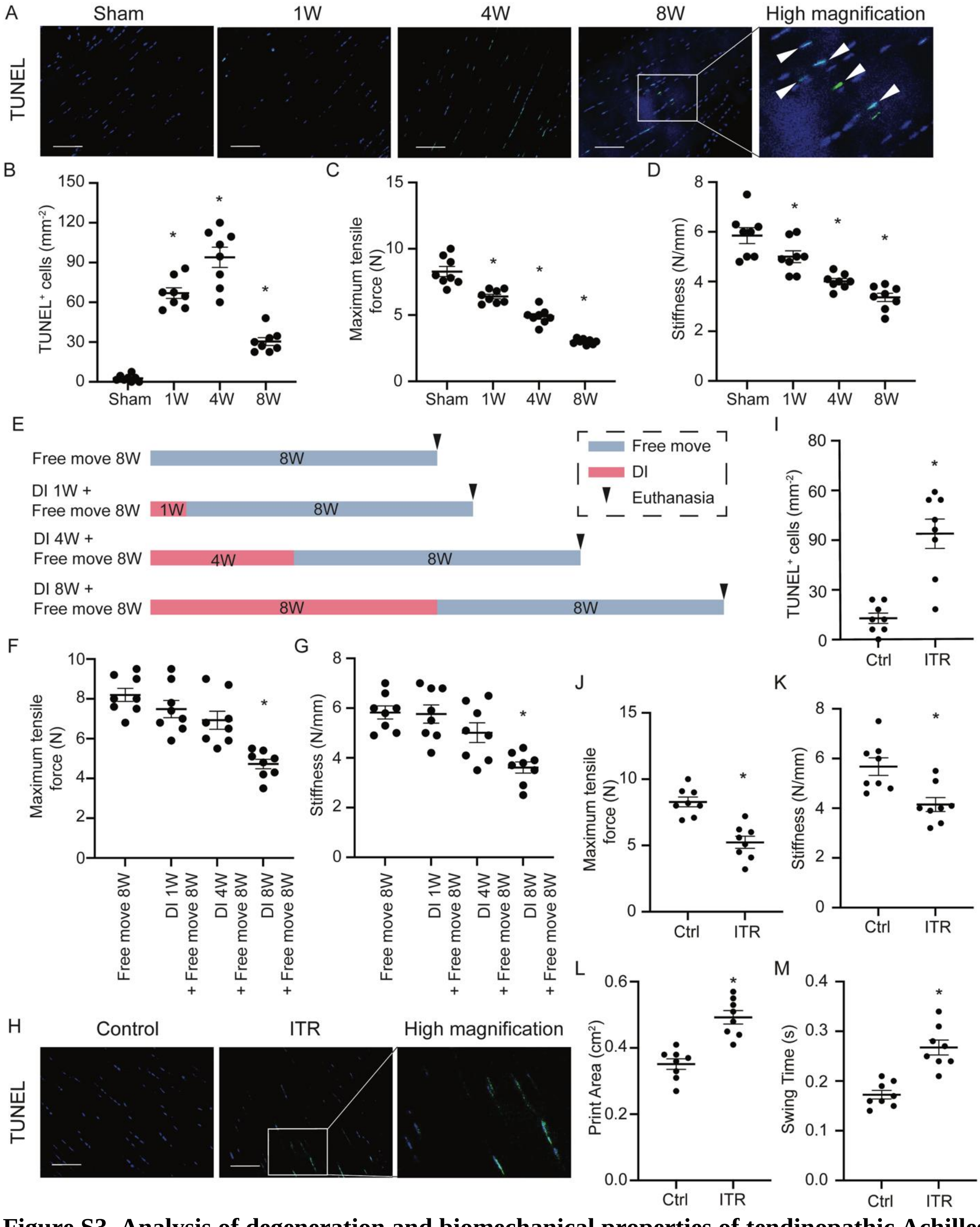

Figure S3. Analysis of degeneration and biomechanical properties of tendinopathic Achilles tendons in DI and ITR mice. (**A**) TUNEL (terminal deoxynucleotidyl transferase dUTP nick end labeling) staining and (**B**) quantitative analysis of apoptotic cells in tendons 1, 4 and 8 weeks

after DI or after sham operation. White arrowheads, $TUNEL^+$ cells. Scale bar: 50 μm (**C**, **D**) Quantitative analysis of (**C**) maximum tensile force and (**D**) stiffness of tendons measured 1, 4 and 8 weeks after DI or after sham operation. All data are shown as the mean ± standard deviation (n = 8 mice per group). *p < 0.05 compared with sham operated mice as determined by one-way analysis of variance to determine significance between groups. (**E**) A schematic diagram of the experiment. DI devices were applied to the mice for 1 week, 4 weeks or 8 weeks. After the last day of DI application, the mice were allowed to move freely in the age for 8 weeks before euthanasia. (**F, G**) Quantitative analysis of (**F**) maximum tensile force and (**G**) stiffness of Achilles tendons 8 weeks after DI was removed. All data are shown as the mean ± standard deviation (n = 8 mice per group). *p < 0.05 compared with free move control group as determined by one-way analysis of variance. (**H**) TUNEL staining and (**I**) quantitative analysis of apoptotic cells in tendons in ITR induced tendinopathy mice and sham control mice. Scale bar: 50 μm. (**J**, **K**) Quantitative analysis of (**J**) maximum tensile force and (**K**) stiffness of tendons after sham or ITR. (**L**) The footprint area and (**M**) the swing time of the hind foot of sham or ITR mice. All data are shown as the mean ± standard deviation (n = 8 per group). *p < 0.05 as determined by unpaired, 2-tailed Student's *t*-test.

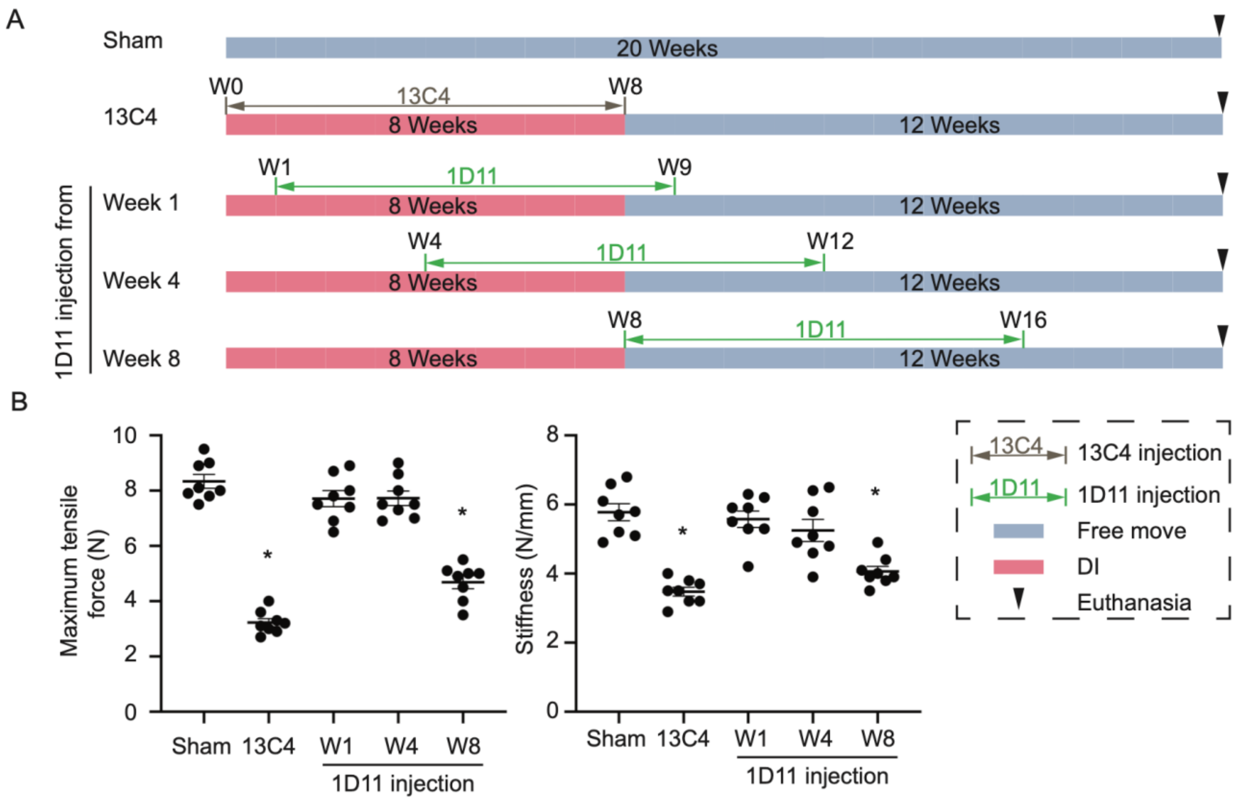

Figure S4. Analysis of biomechanical properties of tendinopathic Achilles tendons after 1D11 injection from different time points.

(**A**) A schematic diagram of the experiment. Mice were treated with 1D11 for 4 weeks from 1 week (W1), 4 weeks (W4) and 8 weeks (W8) after the day DI were applied for 8 weeks and analyzed 12 weeks after DI were removed. 13C4 injection for 8 weeks from the day DI were applied was used as a vehicle injection control. (**B**) Quantitative analysis of maximum tensile force and stiffness of Achilles tendons. All data are shown as the mean ± standard deviation (n = 8 mice per group). *$p < 0.05$ compared with sham group as determined by one-way analysis of variance to determine significance between groups.

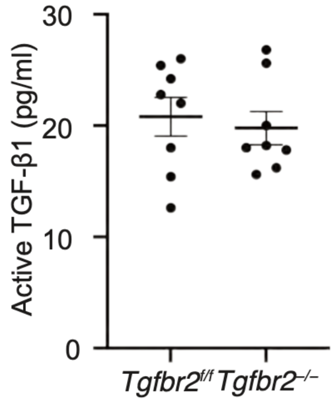

Figure S5. Active TGF-β1 concentration did not change upon deletion of *Tgfbr2$^{f/f}$* in tenocytes.

Tamoxifen was injected to *Scx-creERT2*::*Tgfbr2$^{f/f}$* mice to specifically delete *Tgfbr2* (*Tgfbr2$^{-/-}$*) in the tenocytes. Tamoxifen treated *Tgfbr2$^{f/f}$* mice were used as controls. All data are shown as the mean ± standard deviation (n = 8 mice per group). An unpaired, 2-tailed Student's *t*-test were used to test the differences.

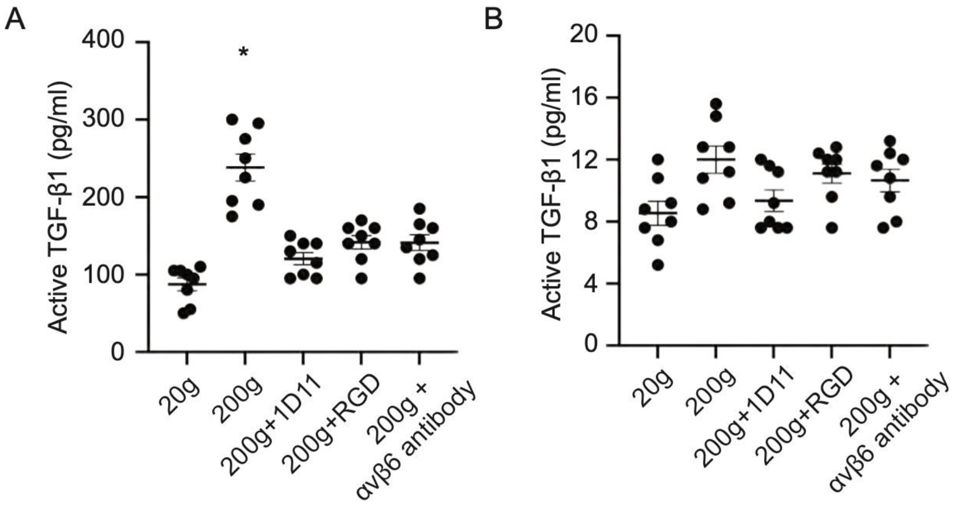

Figure S6. Active TGF-β1 concentration in tendon and culture medium.

(A) Active TGF-β1 concentrations in physiologically loaded tendons and overloaded tendons with vehicle, 1D11, RGD peptide and αvβ6 antibody, and in (**B**) culture medium. All data are shown as the mean ± standard deviation (n = 8 mice per group). *$p < 0.05$ compared with 20g loaded group as determined by one-way analysis of variance.

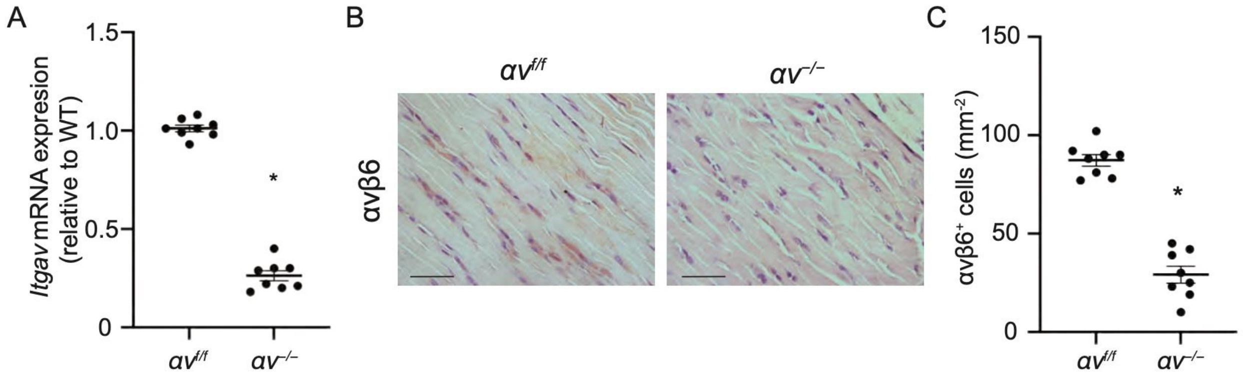

Figure S7. Inducible knockout of *αv* in Scx$^+$ cells significantly decrease the expression of αvβ6 in tendons.

(**A**) Message RNA expression of integrin *αv* (*Itgav*) in Achilles tendons of *Scx-creERT2::αv$^{f/f}$* and their WT *αv$^{f/f}$* littermates 1 week after tamoxifen injection. (**B**) Immunostaining and quantification of tendon sections from *Scx-creERT2::αv$^{f/f}$* and their WT *αv$^{f/f}$* littermates with antibodies against αvβ6 4 weeks after DI. Scale bar: 50 μm. All data are shown as the mean ± standard deviation (n = 8 mice per group). *p < 0.05 compared to *αv$^{f/f}$* mice as determined by unpaired, 2-tailed Student's *t*-test.
